# Osteomyelitis related lameness across animal species: pathophysiology, etiology, epidemiology and experimental model approaches

**DOI:** 10.3389/fvets.2026.1835437

**Published:** 2026-05-26

**Authors:** Andi Asnayanti, Udara Rathnayake, Anh Dang Trieu Do, Ruvindu Perera, Adnan Alrubaye

**Affiliations:** 1 Center for Excellence for Poultry Science, University of Arkansas, Fayetteville, AR, United States; 2 Cell and Molecular Biology Program, University of Arkansas, Fayetteville, AR, United States

**Keywords:** animal models, antimicrobial resistance, lameness, livestock, osteomyelitis, pathogens, poultry, welfare

## Abstract

Osteomyelitis is an inflammatory disorder resulting from the invasion of opportunistic pathogens into bones and bone marrows, causing necrosis and affecting multiple animal groups. It represents one of the most prevalent musculoskeletal diseases, imposing substantial economic losses and raising significant animal welfare concerns across livestock and poultry industries. The pathophysiology of osteomyelitis is characterized by mechanisms of disease development, clinical age, the specific anatomic site in the bone, and structural differences between adults and juveniles. The inability of animals to communicate their discomfort restricts the early diagnosis and treatment of osteomyelitis lameness. Furthermore, osteomyelitis-related lameness is complicated by multiple independent factors, including monomicrobial and polymicrobial infections, latent infections from immune suppression, and emergence of antimicrobial resistance. Chronic osteomyelitis necessitates surgical debridement or additional drug delivery systems in treatment regimen, in addition to antimicrobial drugs. This study proposes a novel framework describing osteomyelitis related lameness linking species-specific patterns with shared pathogenesis. Additionally, this review discusses the pathophysiology, clinical manifestation, epidemiology, and etiology of osteomyelitis related lameness in multiple animal species, particularly livestock and poultry. Furthermore, the development of new antimicrobial compounds and drug delivery to combat osteomyelitis requires an appropriate animal model mimicking the nature of the disease. Thus, this review also presents the pros and cons of existing animal models in osteomyelitis research, including animal welfare concerns of the experimental osteomyelitis models to tackle polymicrobial osteomyelitis.

## 1 Introduction

Osteomyelitis is defined as inflammation of bone and bone marrow, most commonly caused by bacterial infection, which leads to necrosis and destruction of bone tissue, and reactive new bone apposition (1, 2). In the animal production industry, it represents a major economic and welfare challenge, commonly causing chronic lameness, decreased productivity, high culling rates, and carcass condemnations. It can be fatal in severe or untreated cases (3, 4). The disease’s development is driven by complex, multifactorial interactions between pathogen virulence factors, host immune responses, and species-specific anatomical and physiological characteristics, resulting in distinct clinical patterns across livestock and poultry. These clinical symptoms are nonspecific, further delaying the appropriate preventive and curative measures (5). Early diagnosis remains particularly challenging because animals cannot verbalize pain, often complicating disease control and progression to chronic, treatment refractory states (5, 6). Despite intensive research on human osteomyelitis, scientific investigations of osteomyelitis related to veterinary species remain comparatively limited, creating a significant knowledge gap (7, 8). While human studies have elucidated detailed host-pathogen mechanisms, veterinary osteomyelitis is often complicated by production specific pressures such as rapid growth, high stocking density, and frequent traumatic or surgical interventions. These factors drive both monomicrobial and polymicrobial infections, frequently compounded by biofilm formation and emerging antimicrobial resistance (AMR) (4, 5, 9). Furthermore, osteomyelitis-associated lameness exhibits substantial species-specific differences that have not yet been systematically evaluated. Thus, this review aims to synthesize current knowledge on the pathophysiology, clinical manifestations, epidemiology, etiology, and experimental animal models of osteomyelitis-associated lameness, with a particular focus on livestock (cattle, horses, sheep, goats and pigs) and poultry (broilers, turkeys and ducks). By comparing findings across species rather than presenting isolated descriptions, this review identifies key characteristics, highlights persistent knowledge gaps, and proposes an integrative three-axis conceptual framework to guide future research. Furthermore, understanding the main causative agents and clinical manifestations of osteomyelitis in multiple animal species could help to define the most suitable animal model to investigate disease pathogenesis and possible approaches for treating osteomyelitis. Thus, this review also presents the existing experimental Animal Models (AMs) of osteomyelitis and emphasizes the feasibility and welfare concerns.

## 2 Methods

This narrative review was conducted to synthesize current knowledge on osteomyelitis related lameness across livestock and poultry. A systematic literature search was performed using Google Scholar, PubMed and Scopus, with the following search queries: “pathogens,” “bacteria,” “osteomyelitis,” “hematogenous osteomyelitis,” “vertebrate osteomyelitis,” “contagious osteomyelitis,” “osteomyelitis model,” “animals,” “horse,” “cattle,” “goat,” “sheep,” “pig,” “livestock,” “poultry,” “turkey,” “duck,” ‘trauma,” “bacteria,” “*Staphylococcus*,” and “antimicrobial resistance.” Additional studies were identified through complementary approaches beyond the keyword-based literature search. The analysis incorporates English articles published mostly from 1950 until 2026, including case reports. An osteomyelitis dataset from a German-based article was collected through English translation. All unpublished and book chapters are excluded from analysis.

## 3 Pathophysiology, clinical manifestation and therapeutic strategies of osteomyelitis lameness

The pathophysiology and clinical signs of osteomyelitis are shaped by the mechanism of disease development, clinical age, the specific anatomical site involved, and structural differences between adults and juveniles (6, 10). Pathogens reach the infection site through three primary routes: hematogenous transmission leading to hematogenous osteomyelitis (HO); direct contact from the sites of trauma, surgery, and implant, causing contiguous osteomyelitis (CO); and vascular or neurologic insufficiency associated infection (11–13). The disease most commonly affects the vertebrae and intervertebral disks (spondylodiscitis or vertebral osteomyelitis) (14, 15), joints (16), mandible (17), and pelvic bones (18). Pathogens initiate a robust host response upon localization. Osteocytes act as critical sensors of the bone microenvironment, regulating local immunity in bacterial invasions during osteomyelitis (19). They detect bacterial components using pattern recognition receptors (PRRs) such as toll-like receptors (TLRs) at the infection site (20). On the osteocyte’s surface, bacterial pathogen associated molecular patterns (PAMPs) bind to TLRs (21, 22). This binding activates the TLR-MyD88-dependent signaling pathway, resulting in the secretion of proinflammatory cytokines and chemokines (IL-6, TNF-α, CXCL1 and many others) that recruit immune cells (23). This cascade upregulates the receptor activator of nuclear factor-κB ligand (RANKL) expression, which promotes osteoclast-driven bone resorption and attracts macrophages and neutrophils to the infection site (24). The resulting acute inflammation leads to oedema, vascular thrombosis, ischaemia, and rapid tissue necrosis. In chronic osteomyelitis, certain pathogens evade the immune responses and antibiotic treatment by penetrating the sub-micron canaliculi of the osteocyte lacuno-canalicular network (OLCN) of cortical bone, establishing a protected intracellular reservoir of biofilms (25, 26) which perform as a physical barrier against host immune cells and antibiotics (2, 26) facilitating long-term persistence and progression to chronic osteomyelitis (5).

Clinical manifestations of osteomyelitis include lameness, swelling, heat, pain upon palpation, draining tracts, and localized erythema (6). Although viruses and fungi can occasionally cause osteomyelitis, bone infections are primarily caused by bacteria (2). HO is more prevalent in juveniles than in adults due to the presence of metaphyseal vascular anastomoses, which create “hair-pin” loops on the arterial tip with slow blood current, providing a perfect condition for bacterial growth (6). Failure of passive transmission of maternal immunity or concurrent infection, such as septicemia or septic omphalitis, can also be a predisposing factor for HO (27). HO generally occurs in the metaphysis and epiphysis of developing bones, and the diaphysis of long bones in adults (28, 29). Affected individuals typically present acute or subacute lameness, joint effusion, difficulty of standing, and in severe cases; fever, loss of appetite, as well as prolonged recumbency (6, 30). In contrast, CO is more common in adults, emerging from direct infection of the bone at the time of trauma (such as gunshot and sharp related wounds, road traffic accidents, and bite wounds) or surgical implants (5, 31, 32). In the case of trauma, the blood supply to the bone and the surrounding soft tissue can be damaged, reducing local blood flow and compromising the ability of host immune cells to access the affected tissue. Impeded bloodstream also contributes to additional tissue necrosis and dead cells, all of which increase the risk of infection (33). In open trauma or bone fractures, all damaged tissues in the skin, muscle, and bone are exposed to opportunistic pathogens in the environment, amplifying the risks of direct pathogen inoculation (5). Vascular or neurologic insufficiency associated osteomyelitis is less common and typically linked to inadequate blood supply to the lower extremities (2, 34).

**Figure 1:**
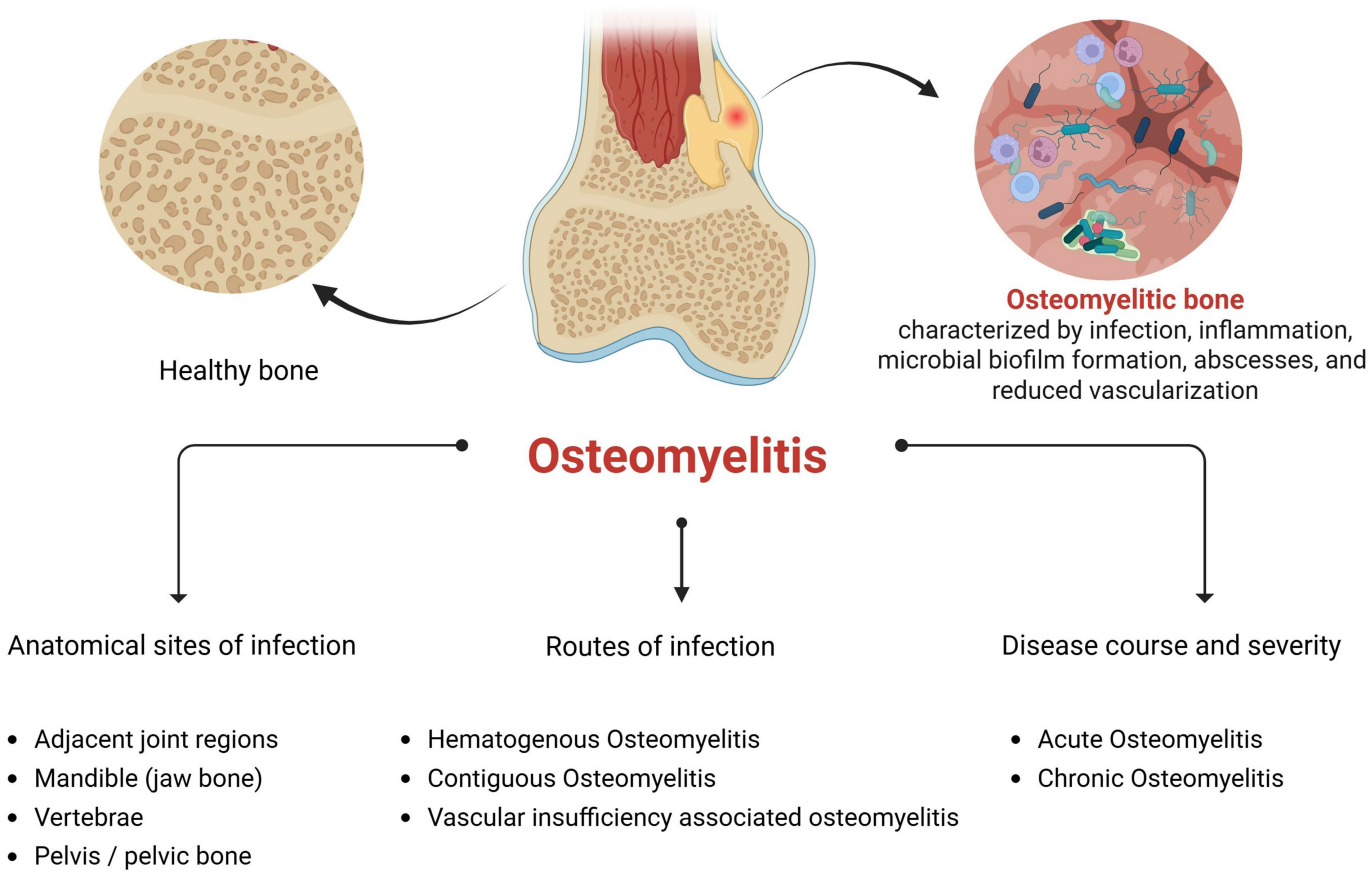
Comparison between healthy bone and osteomyelitic bone, and classification of osteomyelitis. The illustration compares normal bone tissue with bone affected by osteomyelitis. The left side, “Healthy bone” depicts intact bone architecture with normal vascularization and absence of infection. The right panel “Osteomyelitic bone” demonstrates pathological changes including inflammation, biofilm formation, abscess development, and reduced vascular supply. In addition, osteomyelitis is classified based on (i) anatomical site of infection, (ii) routes of infection, and (iii) disease course and severity. This image was created with BioRender (https://biorender.com/).

Osteomyelitis is classified as acute or chronic based on the progression of the inflammatory response and the resulting structural changes in the affected bone, rather than on the overall severity of the disease. Acute osteomyelitis is associated with relatively turbulent blood flow, reduced phagocytic activity, increased susceptibility to injury in the skeletal site, collectively providing favorable conditions for pathogenic escape from the bloodstream (10, 35). The infection induces a severe inflammatory response that leads to congested blood vessels. This vascular compromise prevents immune cells from reaching the infected area effectively and leads to early bone erosion (36). Acute cases develop rapidly (usually less than 2 weeks) (6) with pain, fever, redness, and draining lesions (37–39) which can be relieved with antimicrobial drugs alone (35). Conversely, chronic osteomyelitis is long-lasting (months to years) and characterized with obscured and subtle clinical symptoms—focal swelling, tenderness (37), and bone necrosis (40). This progressive infection of the bone is characterized by a shift from acute neutrophilic infiltration to chronic inflammation. Active infection results in the formation of a sinus to the outer periosteum, creating a periosteal abscess that impairs blood flow and strips the periosteum, forming a sequestrum indicative of a chronic infection (41). Figure 1 illustrates the comparison between healthy bone and osteomyelitic bone, and classification of osteomyelitis.

Osteomyelitis-induced lameness emerges from mechanical instability of bone, hematogenous seeding of bacteria in regions with limited vascularization, and biofilm-mediated chronic inflammation, ultimately destroying bone architecture and causing persistent pain (6). The condition is particularly severe in rapidly growing food producing animals, especially livestock and poultry, where constant bacterial exposure and skeletal development that fails to keep pace with accelerated weight gain contribute to its progression (4). Therapeutic strategies vary significantly with production cycle length, feasibility of individual treatment, and disease stage. To control the infection, new bones (involucrum) are created around the sequestrum (5, 10, 13) but the non-vascularized dead bone remains inaccessible to the immune system and systemic antibiotics (4, 35). When diagnosed early, acute osteomyelitis can usually be managed by targeted antibiotic therapy based on susceptibility testing (6), with broad-spectrum bactericidal antibiotics commonly utilized over treatment durations typically ranging from 4 to 6 weeks or longer. On the other hand, chronic osteomyelitis is much more challenging to tackle compared to acute cases. Therapeutic treatment strategies often require prolonged antimicrobial therapy, additional drug delivery systems (9, 42, 43), or surgical debridement (35, 40, 44) to remove necrotic bone and sequestra, reduce bacterial load, improve antibiotic penetration, and lower the risk of recurrence or further resistance development (35, 40). Antibiotic-resistant pathogens, biofilm formation and emergence of phenotypic variants within pathogens further complicate treatment and promote latent, recurrent infection (6, 27, 40). Treatment approaches also differ across species due to anatomical, physiological, economic, and management factors. Small animals more readily allow for individualized therapy, advanced diagnostics, susceptibility testing, and repeated surgeries with higher success rates (45). In large production animals, however, economic and herd management factors limit treatment options and typically depend more on empirical antibiotics, as well as less invasive approaches (46, 47) though surgical intervention has demonstrated success in several cases (37, 48). A recent case report described successful management of osteomyelitis in cattle using a combination of regional limb perfusion, antibiotics, nonsteroidal anti-inflammatory drugs, and platelet-rich plasma (49). Effective treatment requires an accurate, up-to-date understanding of the microbial agents involved and their antimicrobial susceptibility. Microbiological culture and antimicrobial susceptibility testing are essential for accurate pathogen identification and for guiding targeted therapy, thereby helping to reduce unnecessary or inappropriate antibiotic use (50–52).

### 3.1 Cross-species comparison and conceptual framework

Osteomyelitis-related lameness can be interpreted as a conceptual framework with three interacting axes; (1) mechanical stress on developing or damaged bone, (2) direct inoculation or translocation (hematogenous or contiguous) of opportunistic pathogens, and (3) impaired host immune response leading to chronic infection stage.

Axis (1) predominates in fast-growing poultry. In this context, osteomyelitis-related lameness is best characterized by bacterial chondronecrosis with osteomyelitis (BCO), a disease primarily affecting fast-growing broilers, followed by turkeys and less commonly in ducks (53). BCO results from an interaction between excessive mechanical stress on the developing skeleton and opportunistic bacterial invasion of necrotic lesions in the growth plates and vertebrae (54). In commercial broilers, the body weight increases roughly a 100-fold by the sixth to eighth week of age (54) while the femoral and tibial length only increases approximately four times and mid-shaft diameter about three to five times (55). This disproportion creates excessive mechanical stress and torque on the long chondrocyte columns in the proximal growth plates (54), producing microfractures and osteochondrotic clefts that transect metaphyseal blood vessels, resulting in ischemia and necrosis (55, 56). These lesions are colonized by translocated, hematogenously dispersed opportunistic bacteria primarily from the gastrointestinal tract or the respiratory tract (54). Generally, translocation events arise when stressors such as high stocking density, mycotoxin exposure, or poor nutrition disrupt intestinal tight junction protein functions (55). This disruption increases epithelial permeability, enabling bacteria from acquired and/or resident microbiota to translocate into the bloodstream (55) in a condition often referred to as “leaky gut” (57, 58). Similarly, the respiratory route involves the translocation of aerobically acquired bacteria, which often colonize in the avian air sacs, into the bloodstream causing BCO infection in stressed and immunocompromised birds (54, 59). BCO infections can also affect the fourth thoracic vertebrae (T4), causing bacterial spondylitis—colloquially referred to as “kinky back” (54, 60), sharing similar clinical sign as nonbacterial spondylolisthesis (physical slippage of the same thoracic vertebrae).

Conversely, large livestock animals—particularly adult horses and cattle—are more susceptible via axis (2); contiguous spread of pathogens from surrounding infected soft tissue. Trauma or surgical implants disrupt local vasculature by reducing blood supply to the soft tissue, leading to extensive necrosis and inducing polymicrobial invasion of opportunistic pathogens from the environment (5, 10, 61). This leads to rapid biofilm formation and immune evasion mechanisms that are more common in contiguous pattern of livestock than in the hematogenous pattern typically seen in poultry (4, 61). Small ruminants (sheep and goats) and pigs exhibit patterns of both axis (1) and (2). In juvenile animals, HO prominently occurs in mandibular and vertebral sites, with poor hygiene, failure of passive transfer, and traumatic procedures increasing disease susceptibility (6, 62). In adult sheep and goats, CO is more prevalent, frequently arising from trauma related to foot rots, bite wounds, or chronic abscesses, leading to mandibular osteomyelitis (MO) (63–65). Swine exhibits a distinct profile. Young piglets are more prone to contiguous vertebral and long bone osteomyelitis. Tail biting is also an important contributing factor for piglet osteomyelitis, with tail wounds acting as entry points for pathogenic bacteria (66–68). As such, management practices such as teeth clipping and tail docking are intended to mitigate disease incidence (69, 70). However, they are not sufficient as standalone interventions because they primarily control the symptoms of stress rather than the root causes, and often create their own welfare issues such as acute/chronic pain, stress, tissue damage, infection risk and behavioral changes (71–73).

Axis (3); host immune evasion is evident across multiple species but expressed differently due to distinct bone microarchitecture and immune dynamics of the host. In broilers, this mechanism frequently results in polymicrobial infections localized in the metaphysis of long bones, and as a result these infections exploit permissive environment of necrotic growth plate (19, 25). In large ruminants, equines, sheep, goats, and pigs, deeper bacterial invasion of the OLCN promotes recurrent, polymicrobial infections and the formation of prominent sequestrum. Certain pathogenic bacteria are capable of adapting their morphology to navigate through the narrow canaliculi, evade host immune responses, and persist within the bone matrix (51, 67, 74). These differences in the three-axis framework may explain why poultry associated osteomyelitis is primarily hematogenous and growth plate specific, while livestock osteomyelitis is more related to surgical implants, stronger trauma or management related components (4, 6, 54).

## 4 Etiological agents of osteomyelitis lameness

The characteristics, clinical manifestations, and malignancy of osteomyelitis are determined by the interplay between host factors and pathogen virulence, with the route of infection (hematogenous versus contiguous) exerting a dominant influence on microbial composition and disease pattern (10). Most HO cases, especially acute ones, are typically monomicrobial, while CO cases are frequently polymicrobial (8, 10). This section compares predominant pathogens across livestock and poultry within the three-axis framework of osteomyelitis lameness and highlights key knowledge gaps. Major etiological agents associated with osteomyelitis and related musculoskeletal conditions in livestock and poultry are listed in Table 1.

**Table 1:** Major etiological agents associated with osteomyelitis and related musculoskeletal conditions in livestock and poultry: comparative overview.

Host (age group)	Clinical manifestation and notable features	Key etiological agents	Pattern and primary route	References
Livestock				
Cattle (juveniles)	Joint inflammation, physitis, most commonly occur at the distal metacarpus, metatarsus, radius, and tibia, strong proteolytic activity	*Actinomyces pyogenes, Staphylococcus. aureus*, *Escherichia coli, Enterococcus* sp.*, Bacillus* spp., *Proteus* spp., and *Corynebacterium* spp.	Mostly monomicrobial – Hematogenous	(92, 96, 218)
Cattle (adults)	Mandibular osteomyelitis, lameness, swelling of the right olecranon, Sulfur granules containing phagocytosed bacteria were found in the infection site	*Actinomyces* spp.	Monomicrobial – Contiguous	(95, 97)
Horses (foals)	Bone lesions in the femur, tibia and distal phalanx, and concurrent septic arthritis, high mortality if untreated, failure of passive immunity	*E. coli*, *Proteus* spp., *Enterobacter* spp., *Klebsiella* spp., *Salmonella* spp., *Pseudomonas* spp. *Actinobacillus* spp., *Serratia marcescens, Pasteurella* spp., *Staphylococcus* spp.*, Streptococcus* spp., *Listeria* spp.*, Rhodococcus equi, Clostridia* spp., *Bacteroides* spp., and *Actinomyces* spp.	Polymicrobial – Hematogenous	(101)
Horses (adults)	Lameness, bone, facial or mandibular fractures, septic arthritis, high recurrence after internal fixation	*Corynebacterium pseudotuberculosis, Enterobacteriaceae, Streptococcus* spp., *Staphylococcus* spp.*, Pseudomonas* spp., *Actinobaciflus* spp., *Pasteurella* spp., *Bacillus* spp., *Corynebacterium* spp., *Micrococcus* spp., and *Actinomyces* spp.	Polymicrobial – Contiguous	(106, 109–111)
Sheep and goats (juveniles)	Neonatal polyarthritis, often secondary to omphalitis or tail docking, latent joint destruction	*Fusobacterium necrophorum*, *T. pyogenes*, *S. aureus*, and *E. coli*	Polymicrobial – Hematogenous	(6, 62)
Sheep and goats (all ages)	Mandibular osteomyelitis lesions, foot rot related linked to gingival injury and foot rot	*Streptococcus* spp., *Pseudomonas* spp., *T. pyogenes*, *S. aureus* and *E. coli*	Mostly polymicrobial – Contiguous	(7, 63, 178)
Pigs	Mandibular and vertebral osteomyelitis, carcass condemnations; strong causal link with tail-biting	*T. pyogenes, Staphylococcus* spp., and *Streptococcus* spp.	Polymicrobial – Contiguous	(66, 179, 183, 185)
Poultry				
Broilers (3–6 weeks)	Femoral/tibial BCO, vertebral osteomyelitis, rapid growth is primary predisposing factor, spondylitis (“kinky back”)—colonizes T4 vertebra, spinal cord compression	*Staphylococcus aureus*, *Staphylococcus* spp., *Enterococcus cecorum*, *E. coli*	Mostly polymicrobial – Hematogenous	(54, 60, 89, 219–223)
Turkeys (5–25 weeks)	Osteomyelitis, synovitis, Green-liver syndrome in TOC, transport stress trigger	*S. aureus*, *E. coli*	Polymicrobial – Hematogenous	(137, 140, 168)
Ducks	Femoral and tibial lesions, first confirmation of BCO in Indonesian ducks	*Staphylococcus* spp. and *E. coli*	Polymicrobial – Hematogenous	(141)

### 4.1 *Staphylococcus aureus* virulence and cross species contextualization

*Staphylococcus aureus* is a dominant pathogen in osteomyelitis across species because of its multifaceted virulence arsenal. The pathogenicity and persistence of *S. aureus* are associated with several mechanisms. First, the formation of a micro-capsule or capsular polysaccharide shields *S. aureus* from phagocytosis, thereby promoting its survival within host cells and contributing to persistence in osteomyelitis (75, 76). In addition, the bacterium has a thick cell wall that anchors immune evasion and antiphagocytic properties, which helps it evade host cells’ phagocytic defense. These antiphagocytic properties, called microbial surface components recognizing adhesive matrix molecules (MSCRAMM), are specific cell-surface adhesion molecules that facilitate strong binding to host cells and the extracellular matrix (77–80). Fibronectin-binding proteins and collagen adhesin are examples of MSCRAMM proteins (81). Finally, enzymes and virulence secretions help invade host cell functions (10, 82, 83). *S. aureus* virulence and secretions play essential roles in damaging host tissues, supplying nutrients for bacterial survival and growth, and promoting tissue invasion and colonization, leading to local bone resorption (5, 77, 84). The bacterial regulatory protein Sae is also essential in promoting pathological bone and intraosseous *S. aureus* survival. The Sae-regulated protease aureolysin is a major determinant of the *S. aureus* secretome, inducing osteoblast cell death and bone destruction (85). The formation of *S. aureus* capsule can lead to a dormancy state in the bone, which is a critical mechanism for the development of AMR and latent quiescent osteomyelitis. Once *S. aureus* is engulfed intracellularly in osteoblasts, its intracellular location becomes a harborage from the humoral immune defense of the host and standard prophylactic drugs (77, 86). When local or systemic trauma occurs, the intracellular bacteria may be released from the host osteoblasts’ death. Surgical intervention can reactivate the previously infected bone with dormant bacterial cells, leading to the latency and recrudescence mechanism of clinically quiescent osteomyelitis (77, 86–88). The ability of staphylococci to evade the host immune defense and to withstand antibiotic treatment, leading to recurrent and persistent infections occurring in approximately 40% of patients, has exacerbated patient impairment and proven a major challenge in osteomyelitis treatment (5).

Depending on the dominant axis of three-axis conceptual framework in each species, these mechanisms produce significantly different clinical outcomes of *S. aureus* osteomyelitis. In poultry, *S. aureus* frequently causes polymicrobial infections in proximal growth plate through axis 1. The rapid body weight gain allows hematogenous translocation of *S. aureus*, and the relatively permissive cartilage environment enables intracellular persistence without the need for extensive polymicrobial synergy (89, 90). In livestock, trauma or surgical implants create large avascular niches that favor polymicrobial communities via axis 2, and those pathogens utilize axis 3 through deeper invasion of the OLCN (67, 74). While avian studies demonstrate growth plate cartilage mediated virulence, mammalian studies emphasize OLCN-associated virulence. These cross-species differences in *S. aureus* virulence create a critical knowledge gap and future research should prioritize comparative studies to bridge these gaps and develop species targeted control strategies.

### 4.2 Osteomyelitis causative agents in livestock

Osteomyelitis in livestock seems to involve a wide range of microorganisms. Some cases are monomicrobial infections, yet the vast majority of infections in post-traumatic or post-operative cases involve mixed microbial pathogens (91). Cattle illustrate the clearest age-dependent pathological transition. In juveniles, HO predominates through Axis 1, with metaphyseal vascular loops providing a low-flow nidus for monomicrobial invasion. *Trueperella* spp. (Arcanobacterium) is the leading causative agent of HO in juvenile cattle, while animals under 3 months of age commonly contract *Salmonella* sp. infection (92). A case report of a three-year-old cow exhibiting a purulent fistulous tract presented *Trueperella pyogenes* in the culture of wound exudate (93). *T. pyogenes* is part of the normal microbiota of the skin, upper respiratory, gastrointestinal, and urogenital tracts of animal species and is characterized by strong proteolytic activity. Another case study reported two heifers contracting osteomyelitis: one exhibited septic metacarpal physitis, indicating an HO incidence following bronchopneumonia, whereas the other one demonstrated concurrent serofibrinous arthritis of the metacarpophalangeal joint developing post-trauma. *Escherichia coli, T. pyogenes,* and *Proteus vulgaris* were identified from the culture of the wound swabs (94). A first-lactation dairy cow exhibited a hard, round intumescence on the right olecranon and experienced slight lameness due to *Actinomyces* infection (95). Furthermore, *Actinomyces pyogenes* was discovered as the main etiologic agent with bone infection of the appendicular skeleton, with or without anaerobic coinfection (96). Actinomycotic osteomyelitis and periostitis of the mandible or maxilla are diseases of cattle caused by the Gram-positive, branching filamentous microorganism *Actinomyces bovis. A. bovis* causes granulomatous osteomyelitis (97, 98).

In adult cattle, Axis 2 becomes dominant in VO, following trauma or surgical contamination, resulting in granulomatous mandibular lesions driven *by A. bovis or* polymicrobial infections involving *S. epidermidis, Fusobacterium* spp., *Clostridium perfringens, Salmonella dublin, Pseudomonas* spp., *E. coli,* and *A. fumigatus* (4, 95, 97, 99). This shift highlights a key point of consideration; whether chronicity stems primarily from bacterial virulence factors or from delayed diagnosis in animals unable to communicate pain (4). Furthermore, lameness related to growth plate lesions is a significant issue in cattle. Cattle aged from 2 weeks to 5 years are susceptible to HO, where the most frequently infected sites occur at the distal metacarpus, metatarsus, radius, and tibia (92, 100). Macroscopic lesions in the distal metatarsal physis of lame bulls in northeastern Italy exhibited a purulent and necrotizing physitis, indicating osteomyelitis syndrome, as well as segmental thickening of the physis consistent with physeal osteochondrosis. Lame bulls were likely clinically more severe due to bacteremia, resulting in HO (92).

Osteomyelitis lameness is prevalent in horses (101–103), where foals, especially neonates, commonly suffer from HO (Axis 1) due to inadequate maternal immunity in response to bacteremia (102), while adults are frequently contracted from VO (Axis 2) (104). Gram-negative bacteria, including *Actinobacillus* sp., *Escherichia coli, Klebsiella* sp., *Pseudomonas* sp., and *Salmonella* sp., are more frequently involved in foal infection than Gram-positive organisms such as *Streptococcus* spp. (101). In general, aerobic bacteria, particularly Enterobacteriaceae, *Streptococcus,* and *Staphylococcus*, are commonly isolated from horses (105). *Corynebacterium pseudotuberculosis* infection of the limbs in horses typically results in severe lameness, and the prognosis for survival is poor (106). Furthermore, the incidence of *Rhodococcus equi* infection in horses has dramatically increased, leading to clinical symptoms such as fever, inflammatory leukogram, and eventual lameness (107). In addition, fungal infection can also lead to osteomyelitis in horses. A VO incidence in a horse was reported as a result of *Aspergillus* colonization (104). *Aspergillus fumigatus* was also detected in the synovial fluid culture of a 12-year-old Standardbred gelding experiencing swelling of the right metacarpophalangeal joint (108). In adults, post-traumatic CO (Axis 2) prevails, resulting in highly polymicrobial infections dominated by Enterobacteriaceae and coagulase-negative staphylococci (109–111). Post-operative infection occurred in 192 horses following their internal fixation of long bone fractures and arthrodesis (109), microbiology examination of which revealed 32% Gram-positive, 28% Gram-negative, and 40% mixed bacteria. Polymicrobial infections were also identified in 36 out of the 60 surgically repaired fractures in horses (110). Compared with cattle, equine disease shows greater recurrence linked to deeper OLCN invasion, yet few studies have quantified inter-species differences in osteocyte permissiveness.

Similar to horses and cattle, sheep, goats, and pigs bridge juvenile hematogenous and adult management-driven patterns. The infected joints become hot and swollen because of the hyperaemia and edema of the synovial membrane and joint capsule, which is the most favorable locus of microbial infection. Several HO incidences can be healed; nevertheless, they often lead to latent infection, especially in the large joints of the limbs, leading to destruction of articular cartilage and severe septic arthritis (62). *Fusobacterium necrophorum, Staphylococcus* spp., *E. coli*, *A. pyogenes*, and *S. aureus* are some pathogens frequently isolated from neonatal polyarthritis and osteomyelitis in lambs (62). 67.01% of 250 sheep suffering MO showed mixed cultures from sampling, and 32.98% of the studied sheep showed pure isolates. The genus *Streptococcus* was the most frequently isolated (20.95%), followed by bacteria of the genus *Pseudomonas* (8.50%), *T. pyogenes* (7.26%), *Staphylococcus* spp. (6.63%), and *E. coli* (6.22%) (63). *Streptococcus*, *Staphylococcus,* and *Pseudomonas* are normal residents in the healthy mouth of sheep, indicating that normal microbiota of the oral cavity could be involved in the development of MO lesions when there is an injury of the gingiva, enabling the entry of bacteria to the oral mucosa, which eventually settle locally in bony tissues (112). Farm-level prevalence of MO in culled sheep can exceed 15.5% and is strongly linked to abrasive diets and poor hygiene (64). Osteomyelitis in goats follow similar patterns, with mandibular pyogranulomatous osteomyelitis reported in dairy breeds, often involving opportunistic Gram-positives such as *T. pyogenes* (113). Furthermore, an atypical fatal case of mandibular pyogranulomatous osteomyelitis in an adult female sheep was reported, caused by *Pseudomonas aeruginosa* and *Lactococcus raffinolactis* (114). In swine, osteomyelitis, mediating through Axis 2 and often associated with tail-biting lesions, accounted for 61.03% of carcass condemnations in a Portuguese slaughterhouse study (66). Consistent with this, osteomyelitis has been identified as the primary cause of carcass rejection in Portugal, both in national data (2014–2019) and in García-Díez et al., which reported 38.52% (115, 116). These polymicrobial infections are often dominated by *Staphylococcus* and *Streptococcus* spp. (66).

### 4.3 Osteomyelitis causative agents in poultry

Osteomyelitis in poultry is largely hematogenous and closely associated with rapid growth rate, inducing its vulnerability in proximal femoral and tibial metaphyses, in contrast to the trauma-dominated contiguous pattern in adult livestock (54, 55).

Within poultry species, broilers are the most intensively studied animal group. *S. aureus* was reported as the main causative agent in the first BCO lameness case discovered in chickens in Australia in 1972 (89). As research on BCO lameness progressed throughout the years, multiple species have been associated with BCO progression in broiler chickens, of which *Staphylococcus* spp. remain the most prevalent isolated bacteria (59, 117–119). Most of the opportunistic Staphylococci responsible for osteomyelitis cases in humans were also abundantly found in broiler BCO lameness, including *S. aureus, S. epidermidis, S. hyicus,* and *S. simulans* (90, 120). However, other zoonotic *Staphylococcus* species isolated in broilers with osteomyelitis are rarely present in human cases, such as *S. agnetis, S. cohnii, S. saprophyticus, S. gallinarum, S. xylosus,* and *S. lentus.* Gram-positive Staphylococci are commonly overrepresented in proximal tibial and femoral head necrosis lesions, impairing the locomotion of the birds (90, 121–123).

Another Gram-positive bacterium abundantly isolated from lame birds is *Enterococcus* spp., which is also highly associated with VO in broiler chickens (119, 124). *E. cecorum* and *E. faecalis* are the most isolated *Enterococcus* in BCO lame birds (119, 121, 125). While *E. cecorum* is a common commensal of the gastrointestinal tracts of mammals and birds (126), several outbreaks of arthritis and osteomyelitis lameness in broiler flocks in several countries have nonetheless characterized *E. cecorum* as the main etiological agent (126–129). Typically, *E. cecorum* colonizes the fourth thoracic (T4) vertebra, leading to VO syndrome. In commercial flocks, one out of nine lame birds have been reported to bear VO lesions, especially necrotic degradation of the thoracic vertebrae, leading to complete immobility (18, 54, 130). On the other hand, *Enterococcus* spp. is typically identified in the proximal end of the femur and tibia of the long bones, similar to *Staphylococcus* (89, 130, 131). VO accounts for approximately 20% of osteomyelitis lameness cases in broiler chickens, the typical clinical sign of which involves a hock-sitting posture with the broiler’s legs extended forward in the condition known as spondylitis (128). Several other Gram-positive bacteria have been isolated from broiler BCO lameness cases in lower prevalence, including *Bacillus*, *Corynebacterium*, and *Brachybacterium* (90, 132). In addition to *Staphylococcus* sp. and *Enterococcus* sp., Gram-negative bacteria, *E. coli and Salmonella* spp., have also been identified in the culture of blood and BCO lesions of lame birds from three commercial broiler production houses in Arkansas. These bacterial isolates were geographically dependent and varied among the production sites (133).

In turkey, osteomyelitis is characterized by avascular necrosis with sequestration and dyschondroplastic cartilage, with spontaneous fracture and collapse of the femoral head or fracture through femoral metaphyses (femoral necks). Necrosis usually occurs beneath the growth plate in the metaphyses (134). Etiology of lameness in turkey involves a wide range of microbial infections from various species, including *Histomonas meleagridis* (135), *Mycoplasma synoviae* (136), *Actinomyces pyogenes* (137, 138), and Turkey arthritis reoviruses (139). Somewhat expectedly, *S. aureus* and *E. coli* are also known as causative agents of turkey green-liver osteomyelitis complex (TOC), a typical osteomyelitis primarily implicating adolescent male turkeys with green discoloration of the liver. The birds suffering from TOC lesions have a significantly decreased cell-mediated immunity response, allowing for the occurrence of opportunistic bacterial infections (135). In a TOC case involving green livers, it was found that the associated hyperplasia of bile ducts, dilation of sinusoids, and pigment-containing Kupffer’s cells presented pleomorphic Gram-variable coccobacilli from bone and liver cultures (136). A study of 149 turkeys aged 5–25 weeks exhibiting osteomyelitis and synovitis syndromes also presented *S. aureus* and *E. coli* isolates in their bone lesions. The sites of infection included the proximal femur, proximal tibia, distal femur, and proximal metatarsal of the leg bone, yielding soft, yellow, and focal lesions. In addition, *P. aeruginosa* was also identified in the two cases in this report (140).

In addition, a *Yersinia pseudotuberculosis* outbreak was diagnosed in a male turkey flock in Finland. The bacterium was isolated from the femoral and tibiotarsal bones of the joints of six lame turkeys aged from 11 to 12 weeks old. During pathological examination, multiple lesions were found in the metaphyseal and physeal areas of the femurs, tibiotarsi, and tarsometatarsi, with multifocal to coalescing mixed heterophilic/granulomatous necrotizing osteomyelitis. *Y. pseudotuberculosis* is a rare zoonotic bacterium, which typically causes enteritis in humans and sudden death in animals (62).

Only a limited number of systematic evaluations have been conducted in ducks. In 2025, a survey of commercial flocks in Indonesia reported a 44% prevalence of femoral and tibial BCO lesions, predominantly associated with *Staphylococcu*s spp. and *E. coli* (141). This study confirmed that rapid growing meat type ducks follow the same mechanical translocation pathway as broiler chicken, with potentially lower clinical lameness expression due to shorter production cycles and different housing. The findings extend the poultry BCO paradigm beyond chickens and turkeys and highlight the need for species-specific surveillance in expanding duck industries.

## 5 Epidemiology of osteomyelitis-related lameness across livestock and poultry

Osteomyelitis-related lameness has emerged as a major disease across livestock species and poultry, influenced by management practices and host-pathogen interactions. Lameness is characterized by abnormalities in gait and posture related to the skeletal system, causing pain or discomfort and leading to substantial negative impacts on animal welfare, production efficiency, and economic viability (142, 143). Common conditions causing lameness include degenerative joint disease, osteochondrosis, physitis (epiphysitis), exertional rhabdomyolysis, and trauma, which can also be a predisposing factor to the development of severe osteomyelitis in domestic animals (144). Recent systematic reviews reveal consistent patterns that highlight management and environmental drivers as the dominant risk factors over genetics in most species. However, prevalence data vary widely due to differences in study design, diagnostic criteria, geography, and production systems.

### 5.1 Osteomyelitis-related lameness in livestock

In livestock, lameness is generally defined as the manifestation of skeletal pathologies causing uncomfortable or painful standing and walking gait in affected animals, resulting in impacted welfare and reduced productivity (142, 143). Osteomyelitis-associated lameness causes tremendous economic losses and animal welfare issues across the livestock farming industry.

In cattle, lameness is widely regarded as a major welfare issue, exacerbated by a shortage of early diagnostic systems (145). Lameness demonstrates a global point prevalence of 17–35% in dairy herds, with meta-analyses in Northwest Europe reporting a weighted mean prevalence of 28% (95% CI 23–33%) using lenient scoring, 23% (95% CI 18–29%) and 9% (95% CI 5–12%) for severe lameness cases using conservative and lenient scoring, respectively (146–149). A broader global literature review of 53 studies which spanned a 30-year period (1989–2020) and included herds from six continents, with the majority from Europe and North America estimated a mean prevalence of lameness 22.8% (median 22.0%) and mean prevalence of severely lame cows 7.0% (median 6.5%) with substantial heterogeneity attributable to housing type and scoring method (150). Generally, lame cows have an increased risk of early culling. Severe lameness can also lead to carcass condemnation, even if the animal remains fit for transport or slaughter (143, 144). Total or partial carcass condemnation occurs when post-mortem inspection identifies potential food safety risks, such as systemic infection, localized inflammation, or other hazards associated with the lameness (144). VO is rarely found in juvenile cattle (151), while HO predominates in juvenile cattle (threefold higher than post-traumatic cases), while trauma becomes the leading cause in adults (96, 152). Another report presented that trauma is one of the main predisposing factors of osteomyelitis in cattle (152). Hindlimb lameness is common in dairy cattle, while hip dislocation, upward luxation of the patella, and wounds are predominant lameness determinants in buffaloes (144).

Key risk factors can be organized into a clear hierarchical pattern; with management-related concerns including concrete flooring, poor bedding, lack of pasture access, high parity, low body condition score ranking the highest, followed by environmental conditions, while breed or genetic factors play a comparatively minor role (144, 149, 153). These factors are driven by both causal mechanisms; mechanical overload on hooves leading to secondary osteomyelitis and associations; increased risk of death, reduced milk yield and reproductive performance in lame cows (154, 155). Overall, extensive economic losses in cattle arise from decreased milk production, prolonged calving intervals, high treatment costs (~$75 per cow/year in the US), premature culling, and carcass condemnation (144, 147, 156).

Similar to cattle, horses frequently develop osteomyelitis, where HO is more prevalent in foals and CO is predominant in adults following fractures or implants (6, 102). According to previous reports, lameness incidence ranges from 21 to 33% depending on the population and region (13, 144, 157), with slightly higher involvement in forelimb than hindlimb (13). Musculoskeletal problems in horses are costly to treat (158), while environmental conditions (temperature and humidity) and management practices contribute to disease severity, making this multifaceted disease difficult to control in equines (144, 159).

Small ruminants and pigs exhibit a trend that bridge HO and CO, in juvenile and adult populations, respectively. In sheep and goats, lameness incidence reaches approximately 10% in 2006 in UK pasture systems (160), resulting in an estimated £24 million financial loss (161). The incidence of lameness in sheep is high during wet months due to foot rot and traumatic injuries that progress to mandibular or sternal osteomyelitis (35, 160, 161). Osteomyelitis is considered the main cause of condemnation of pig carcasses in Portugal, leading to significant economic losses. One study reported that it accounted for the rejection of 61% of the carcasses analyzed. Among lesions located in the anterior region, 94.78% corresponded to cases of mandibular osteomyelitis (66).

### 5.2 Osteomyelitis-related lameness in poultry

In poultry, osteomyelitis manifests primarily as BCO, the leading cause of lameness in fast-growing broilers (89). Unlike livestock, rapid growth is the main cause of lameness in poultry. Management practices and environmental factors act as inducers rather than primary causes (117, 162, 163). Severe lameness leads to feed and water deprivation (117, 164). Substandard hygiene practices and high bird density of commercial farms have been shown to induce the vulnerability to pathogenic infection in commercial broiler flocks (117, 162, 165). BCO lameness is the most widespread musculoskeletal disorder, causing animal welfare issues and substantial financial losses, accounting for global flock level losses range from 10 to 30%, with post-mortem BCO lesions; 15% (Bulgaria), 19% (Europe) and 28% (Australia) (134, 165–167). The rate of bird culling due to BCO lameness in commercial farms ranges from 0.5–4%, accounting for approximately $100 million in annual economic losses in the United States (117, 164).

Osteomyelitis is also prevalent in species other than broilers. A study examining 149 lame turkeys aged from 5–25 weeks revealed that 56% of lame birds suffered from osteomyelitis and synovitis (140). In 15-week-old male breeding turkeys, a TOC disease outbreak likely induced by transport stress (140) caused greenish livers, swollen joints, soft-tissue necrosis, and osteomyelitis in the proximal tibia (168). Recent evidence from commercial duck production systems in Indonesia has confirmed the presence of BCO, with 44% birds showing femoral and tibial lesions (141). These findings highlight the need for expanded research on osteomyelitis and BCO across a wider range of poultry species and in underrepresented geographic regions, particularly in emerging poultry-producing countries.

### 5.3 Hierarchy of risk factors and knowledge gaps

Comparative analysis across species highlights a consistent hierarchy of risk factors. In most livestock systems, management and environmental drivers—including housing condition, flooring, stocking density, hygiene, and pasture access—contribute significantly toward osteomyelitis incidences, whereas genetic selection for growth rate plays a leading role in poultry (117, 148, 150). Methodological variation including locomotion scoring in livestock (169, 170), visual assessment (gait scores and bone scoring during necropsy) in poultry (171, 172) and geographic bias toward intensive systems determine the differences in lameness prevalence estimates. While the associations between lameness and reduced productivity are well documented, fewer studies have been able to establish direct causality due to the complex, multifactorial origin of the condition. The absence of standardized, cross-species longitudinal studies that quantify the relative contribution of genetics, management concerns and environmental factors for osteomyelitis lameness represents a critical knowledge gap. Filling this gap would enable targeted interventions across different production systems while advancing animal welfare and reducing the utilization of antimicrobial agents (4, 8, 149).

## 6 Experimental animal models for investigating osteomyelitis

Investigating the pathogenesis of osteomyelitis, alongside the development of prophylactic drugs and effective treatments to mitigate infection, necessitates reliable and clinically relevant experimental AMs. A broad range of animals such as rats, mice, rabbits, avian species, dogs, sheep, goats, and pigs has been employed in osteomyelitis experiments (5, 8, 37). AMs offer a more reproducible, controlled environment that can be manipulated to reflect different disease presentations, compared to clinical cases (173). These models can be broadly classified into small animal models—SAMs (rats, mice, rabbits, and avian species) and large animal models—LAMs (dogs, sheep, goats, and pigs) (8). Each category offers distinct advantages and inherent limitations in replicating human disease features such as bone anatomy, surgical scalability, infection chronicity, and polymicrobial dynamics. Both models fail to fully encompass all aspects of osteomyelitis, requiring careful selection based on research objectives (45). This section aims to critically compare LAMs and SAMs, with particular emphasis on the under-reviewed avian model as a valuable alternative model to evaluate measures controlling polymicrobial osteomyelitis. Table 2 summarizes key strengths, limitations and welfare considerations based on reviewed literature.

**Table 2:** Comparative characteristics of experimental animal models used in osteomyelitis research.

Model category	Key advantages	Major limitations	Welfare considerations	References
LAMs	Human-scale anatomy and implants, similar bone mineral density and vascular supply, realistic surgical procedures and chronic biofilm production	High cost, large housing needs, long production cycles, ethical restrictions, lower throughput	Higher ethical restrictions, requires strong analgesia and early endpoints	(8, 37, 45, 48, 183, 184)
SAMs	Low cost, short production cycles, genetic tractability, high throughput and mechanistic depth	Small size limits human-scale surgery, often needs high dose of inoculation, limited polymicrobial mimicry, variable bone remodeling	Moderate, invasive procedures common, amenable to refinement via imaging	(37, 45, 174, 198)
Avian model	Natural polymicrobial infection without artificial inoculation, low cost, short production cycle, high disease incidence, scalable for large trials	Differences in bone physiology growth-plate dominance and human long-bone implants	Excellent advancement of 3Rs, non-invasive induction, reduction via large groups, refinement via lameness scoring	(118, 121, 209, 224, 225)

### 6.1 Common methods to induce osteomyelitis in animal models

In AMs, three primary methods are used to induce the bacterial infection: post-traumatic, implant-associated, and hematogenous induction (45, 48). These methods are designed to manipulate disease presentation and test new therapeutic interventions across different species. These induction models generally utilize bacterial strains that exhibit strong affinity for bone, most notably *S. aureus* (174). Post-traumatic induction involves creating a defect (fracture, hole, open wound) in the bone, followed by inoculation of pathogenic bacteria, often mimicking trauma, injuries or surgical wounds. In this model, sclerosing agents are used to cause vascular necrosis, or contaminated foreign bodies are directly administrated into the wound to improve infection rates (175). This model helps to understand how bacteria can spread along with the bone tissue and through the bloodstream. Post-traumatic induction methods reliably produce acute/chronic infection, but are not effective in larger species due to their variable reproducibility (176). In implant-associated induction, a contaminated implant (Kirschner wire, screw, nail, plate) is inserted into the bone, sometimes with adjacent soft-tissue to induce the infection. This model focuses on the formation of biofilms that adhere to the implant surface, creating a protective matrix and making them resistant to antibiotics. These implants can also serve as antimicrobial delivery vehicles against bacterial colonization. Finally, in the hematogenous induction model, pathogenic bacteria are directly injected into the bloodstream, usually through the lateral tail vein, to simulate seeding into the bone marrow (8). This approach has several limitations, including high bacterial loads required to ensure reliable localization in the bone, variable reproducibility, and high disease complication rates (37, 45, 173). These limitations arise primarily from the non-specific nature of the model (177).

### 6.2 Large animal models

Experimental osteomyelitis has been developed in large animals such as dogs, swine, sheep, and goats as their anatomical and physiological features substantially resemble those of humans, particularly in bone size, mineral composition, vascular supply, and biomechanical properties, allowing for evaluation of new surgical approaches or therapeutic strategies (178–181). For example, it has been reported that canine and porcine models have mineral proportions in femoral cortical and lumbar trabecular bone tissues equal to those of humans (91, 182). Curtis and Kaarsemaker worked on an open fracture goat model and a new model of chronic osteomyelitis in sheep (183, 184). Similarly, the swine model has been the most widely used in the research of HO (91, 177, 183–185), chronic mandibular osteomyelitis (186), tibial implant-related osteomyelitis (62, 101, 187, 188), and traumatic tibial osteomyelitis (189). Typically, osteomyelitis lesions are induced by intravenous or intra-arterial inoculation of bacteria, with or without additional trauma. The HO model was introduced by intravenous (IV) administration of *S. aureus* through a lateral ear vein, resulting in acute, suppurative pneumonic and osteomyelitic lesions in the long bones and the costochondral junctions of ribs (37, 91, 179). This approach usually requires the induction of artificial bone necrosis for subsequent development of macroscopic lesions. The next inoculation method is intra-arterial (IA) inoculation, resulting in the subsequent development of local HO (91, 177, 183, 185). This method is particularly useful for studying the hematogenous spread of pathogens and the rapid establishment of localized bone infections, by delivering pathogens directly into the systemic circulation.

LAMs are ideal tools in investigating the pathogenesis of chronic osteomyelitis, replicating human-scale surgical challenges, and chronic biofilm persistence (37, 45). Under clinically relevant settings, they allow testing of surgical revision strategies, antibiotic-loaded biomaterials, and local drug delivery systems. Additionally, LAMs are crucial for transitional research (190, 191) as their size permits the use of repeated clinical imaging technologies (CT and MRI) for non-invasive, longitudinal monitoring of disease progression and therapeutic efficacy (192, 193). However, these advantages come with substantial drawbacks (45). LAMs are costly to maintain, require specialized housing conditions and surgical expertise, and involve long-term production cycles. In terms of technical practices, osteomyelitis induction via direct bacterial inoculation, either through IV or intra-arterial administration, is time-consuming, difficult to handle, and sometimes requires additional necrosis induction (91). In addition, ethical considerations are also strict, particularly when using companion animals such as dogs, making them less suitable as experimental animals. Moreover, many LAMs are predominantly applied for monomicrobial infection models, limiting their ability to replicate the polymicrobial complexity commonly observed in natural clinical settings. Overall, while LAMs are suitable for study human-scale and biofilm-associated osteomyelitis, their high cost, housing conditions, ethical concerns and technical complexity limit the large-scale utilization in animal studies (45, 173).

### 6.3 Small animal models

Small animals, such as mice, rats, guinea pigs, and rabbits are more frequently used to induce experimental HO compared to LAMs due to their affordability, ease of handling, and suitability for high-throughput experimentation (91). These SAMs are widely used for screening antimicrobial compounds, vaccines, and anti-biofilm strategies in a controlled and reproducible manner. The availability of transgenic strains and immunological tools facilitate in-depth investigation of host-pathogen interactions, immune responses, and molecular pathways of infection progression. Numerous studies have used murine models to study chronic osteomyelitis (194), acute osteomyelitis (195), fracture osteomyelitis in the femur (196), implant-related osteomyelitis (197) and antimicrobial therapies. Similarly, rabbits have also been intensively employed as a model to investigate bacterial osteomyelitis of the tibia (198), chronic osteomyelitis (199), post-traumatic osteomyelitis (107), and antibiotic treatment for osteomyelitis (200). Acute and chronic experimental posttraumatic osteomyelitis was also studied in guinea pigs (11) and mice (201). The induction of experimental osteomyelitis using these animals was mostly performed by bacterial inoculation with or without mechanical trauma or fracture, and implant insertions (37).

Compared to LAMs, SAMs are much more economically efficient in terms of animal housing and production cycles, as well as the capability to induce several characteristics of osteomyelitis, including HO (202), VO (203), chronic osteomyelitis (199), and implant-related osteomyelitis (100), making them highly suitable for the studies of prophylactic therapies to control osteomyelitis. Despite these strengths, SAMs have important translational limitations. The size of small animals restricts the use of clinically relevant implants and surgical procedures, thus reducing their applicability to human orthopedic scenarios (8, 84, 182). Furthermore—like LAMs—most SAMs rely on high-dose, monomicrobial inoculation to induce infection, which does not accurately reflect the polymicrobial and opportunistic nature of many clinical osteomyelitis cases (37, 182). Differences in bone physiology, immune responses, and healing rates further complicate the applicability of this model in translational research (37, 45).

### 6.4 Avian model

The avian model was first developed by Emslie to facilitate acute HO from inoculation of bacteria into a wing vein (204, 205). This model successfully induced microscopic and macroscopic osteomyelitis after 12 and 24 h of post bacterial inoculation. In current literature, several studies of osteomyelitis induction using poultry species, particularly broilers, have been conducted. Osteomyelitis in broilers, commonly recognized as BCO lameness, can be experimentally introduced by direct bacterial inoculation or naturally acquired lameness using a wire-floor model (206, 207). Developed by Wideman et al. in 2012, the wire-floor model remains a gold standard in highly reproducible induction of BCO lesions and associated lameness as the broiler ages. Rearing birds on wire flooring can induce natural polymicrobial infection without direct bacterial inoculation, which was hardly achieved in existing AMs until that point (118, 206, 208). Fast-growing broiler chickens have an inherent risk of developing osteomyelitis diseases because of the non-synchronized body weight gain and structural bone maturation (117).

In this model, pens with wire flooring are constructed with feeders and water pipelines placed in opposite directions to facilitate bird movement. The wire size is 5 cm x 5 cm, and the flat wire panels are elevated to a 30 cm on bricks, allowing the feces to pass through and aggregate underneath the wire panel (54, 118, 206, 208). The wire floor triggers mechanical stress on susceptible joints while the animal moves to access the feed and water sources strategically placed in opposite ends, promoting micro-fractures in the proximal epiphyseal-physeal growth plates of these joints. Concomitant exertion of mechanical forces on vulnerable bones and physiological stress in fast-growing broilers induce immunosuppression, intensifying pathogen infection and leading to leaky gut syndrome, subsequently resulting in high bacteremia and eventually bacterial colonization of the bones (58, 59, 118, 162, 209, 210).

The avian wire-floor model offers additional advantages. This model can induce BCO lameness rates of up to 85% in broiler chickens and has been successfully used to evaluate the efficacy of feed additives and multivariant eBeam vaccine in reducing BCO lameness in broiler chickens (132, 208, 211–213). The experimental osteomyelitis in broiler chickens is reproducible and low-cost, with a short production cycle, and provides efficient research space. Moreover, this model can be applied to large-scale clinical trials and is capable of creating natural polymicrobial infections without direct bacterial inoculation, making it suitable to investigate a large spectrum of new antimicrobial compounds and other applicable treatments, which are hard to perform in any other AMs (55). As improvements in animal welfare, this model reduces the need for invasive procedures and artificial infection, thereby refining experimental protocols. Furthermore, its high disease incidence allows statistically robust outcomes with fewer experimental replicates. Early lameness scoring systems and non-invasive monitoring further enhance refinement by enabling timely intervention and minimizing animal suffering (54, 118).

## 7 Discussion

The conceptualization of osteomyelitis-related lameness along three interacting axes—mechanical stress, pathogen entry, and immune evasion—provides a unifying framework to interpret species-specific disease patterns while highlighting shared pathogenic principles. These three axes converge dynamically, with their relative contributions shaped by host physiology, management practices, and environmental exposures, rather than acting independently. This integrative perspective helps to explain why osteomyelitis remains as a rapidly progressive, high-incidence condition in some systems such as broiler production, yet sporadic and typically chronic in livestock production. Collectively, this three-axis framework not only clarifies species-specific disease mechanisms but also has practical implications for intervention strategies. In poultry, reducing mechanical stress through genetic selection, nutritional optimization, and management changes may be as critical as antimicrobial approaches. In large animals, preventing contamination and improving surgical practices are essential to mitigate disease incidences. Across all production systems, targeting immune evasion strategies; such as disrupting biofilms or enhancing host immunity remains a key challenge. Future research integrating these three axes across experimental models may yield more effective, context-specific strategies to mitigate osteomyelitis and its associated lameness.

Osteomyelitis typically develops only after substantial microbial invasion, significant post-traumatic bone damage, or contaminated foreign implants (214). Regardless, various factors of etiological agents and clinical manifestations of osteomyelitis pose a significant challenge to its treatment (37). The complex interplay of etiological agents in osteomyelitis development, whether monomicrobial or polymicrobial complicates therapies targeting osteomyelitis in animals. Generally, monomicrobial osteomyelitis, particularly HO, can be treated with antibiotic regimens, as the choices of antibiotics are straightforward against a singular pathogenic species. In contrast, polymicrobial osteomyelitis remains a great challenge in treatment. The fact that polymicrobial osteomyelitis involves a wide range of microorganisms, and limits the options for prophylactic drug treatments, as the choices of large-spectrum antibiotics are scarce. Furthermore, latent infection also magnifies the complications of polymicrobial infection, requiring a large spectrum of antimicrobial action modes, prolonged antimicrobial treatments, or necessitating higher doses of antimicrobial applications (215). Moreover, chronic osteomyelitis worsens treatment complexity as surgical debridement is necessary in addition to antimicrobial drug applications. Thus, constant innovative approaches to develop novel, effective diagnostic and therapeutic measures are highly essential to disease treatment.

Improving and presenting new strategies for osteomyelitis diagnosis, prevention, and curative treatments requires relevant AMs for *in vivo* investigations (4, 8). The choice of AM will affect the quality of alternative surgical or treatment approaches and clinical trials evaluating therapeutic strategies, including new antimicrobial compounds and a drug delivery system to reach infection sites (4). In the context of osteomyelitis pathology, each AM possesses unique features to experimentally induce the disease, eliciting specific characteristics of animal diseases being investigated. Thus, the advantages and drawbacks of each AM, including osteomyelitis induction strategies, should be meticulously examined before choosing an *in vivo* experimental model. Up until now, studies of experimental osteomyelitis employing LAMs and SAMs have successfully reproduced monomicrobial infection of osteomyelitis, leading to the advancement of strategies targeting monomicrobial osteomyelitis. Nonetheless, the study of polymicrobial infections and associated experimental models remain elusive, hard to reproduce, and present formidable therapeutic challenges. The results achieved with the avian model have thus shown significant promise in the induction of naturally occurring experimental polymicrobial osteomyelitis. Alongside LAMs and SAMs, the avian model can potentially be utilized to develop and assess the efficacy of new compounds covering a large spectrum of pathogens, adding to the repertoire of research tools for in-depth investigation of osteomyelitis treatment management and drug therapies.

Finally, all experimental animal studies are performed under strict ethical oversight of institutional animal care and use committee (IACUC) policies and national guidelines implementing the principles of Replacement, Reduction, and Refinement (3Rs) (216, 217). Although these AMs may result in temporary discomfort, refinement practices including optimized anesthesia protocols and postoperative analgesia techniques are routinely applied to mitigate pain and stress. The responsible utilization of ethically governed AMs provides fundamental knowledge necessary to improve animal welfare. In summary, these AMs are primarily designed to establish preventive strategies, improve management practices, and enhance long-term welfare in both experimental and commercial animal populations.
